# Participation in antimicrobial stewardship among intensive care unit nurses: a cross-sectional study based on the COM-B model

**DOI:** 10.3389/fpubh.2026.1922847

**Published:** 2026-09-17

**Authors:** Xiao Dang, Hao Liu, Hong Du, Lan Zhang, Lijingzi Wang, Juan Han, Jianping You, Wen Kang, Cong Ren, Pei Li

**Affiliations:** 1 Department of Infectious Diseases, Tangdu Hospital, Fourth Military Medical University, Xi’an, China; 2 Department of Nursing Affairs, First Affiliated Hospital of Xi’an Jiaotong University, Xi’an, China; 3 Department of Infectious Diseases, The First Affiliated Hospital of Army Medical University, Chongqing, China

**Keywords:** antimicrobial stewardship, behavior, intensive care units, latent profile analysis, nurses, self-efficacy, structural equation modeling

## Abstract

**Objective:**

This study aimed to investigate antimicrobial stewardship participation and its associated factors among intensive care unit (ICU) nurses based on the Capability, Opportunity, Motivation, and Behavior (COM-B) model.

**Methods:**

A cross-sectional survey was conducted among ICU nurses from 11 hospitals across China in March 2026. Data on demographic and occupational characteristics were collected using a general information questionnaire. Antimicrobial stewardship participation and its influencing factors were assessed using the ICU Nurses’ Participation in Antimicrobial Stewardship Behavior and Influencing Factors (ASP-BF) Scale, and nursing professional self-efficacy was measured using the Nursing Professional Self-Efficacy (NPSE) Scale.

**Results:**

A total of 497 ICU nurses were included. The mean scores were 48.80 ± 9.86 for capability (possible range: 13–65), 38.42 ± 10.60 for opportunity (possible range: 11–55), 82.55 ± 16.86 for motivation (possible range: 23–115), 59.94 ± 13.57 for behavior (possible range: 16–80), and 29.60 ± 4.45 for NPSE (possible range: 7–35). Structural equation modeling (SEM) showed that capability (*β* = 0.41), opportunity (*β* = 0.84), and NPSE (*β* = 0.77) were positively associated with motivation, and motivation was positively associated with behavior (*β* = 0.75; all *p* < 0.001).

**Conclusion:**

ICU nurses had generally favorable levels of capability, opportunity, motivation, behavior, and NPSE in relation to antimicrobial stewardship participation. Motivation was directly associated with participation behavior, while capability, opportunity, and NPSE were associated with behavior through motivation. In addition, antimicrobial stewardship training was associated with better participation behavior. Future interventions should focus on strengthening institutional support, improving multidisciplinary collaboration and workflow conditions, and providing structured training to enhance nurses’ participation in antimicrobial stewardship.

## 1 Introduction

Antimicrobial resistance (AMR) has emerged as a critical global public health threat. The most recent global assessment estimated that in 2021 there were 1.14 million (95% UI 1.00–1.28) deaths attributable to bacterial AMR and 4.71 million (4.23–5.19) deaths associated with bacterial AMR worldwide. More than 1 million deaths have been attributable to AMR every year since 1990, and under current trends the annual burden is projected to rise to 1.91 million attributable and 8.22 million associated deaths by 2050 (1). The inappropriate and excessive use of antibiotics in healthcare settings is widely recognized as a principal driver of this phenomenon, facilitating the emergence and spread of resistant pathogens (2). Intensive care units (ICUs) represent a particularly high-risk environment: infections are highly prevalent, and critically ill patients frequently require early initiation of broad-spectrum antimicrobial therapy (3). Antimicrobial exposure is consequently more intensive in the ICU than in other inpatient settings, and ICU patients are reported to be five to ten times more susceptible to healthcare-associated infections than the general hospitalized population (4). Such high levels of antimicrobial use substantially increase the risk of multidrug-resistant organism emergence and transmission (5). Antimicrobial stewardship programs (ASPs) have therefore been widely promoted as a means to optimize antimicrobial prescribing and contain the further development of resistance (5, 6).

ASPs aim to ensure appropriate antimicrobial therapy with respect to drug selection, dosage, route of administration, and treatment duration (7). Evidence from well-implemented stewardship programs indicates that such interventions can reduce unnecessary antibiotic prescribing, improve clinical outcomes, and limit the spread of resistant organisms (6, 8). Effective stewardship is inherently multidisciplinary, requiring coordinated collaboration among physicians, pharmacists, microbiologists, infection control specialists, and nurses (9). Although stewardship initiatives have historically been led by physicians and pharmacists, growing attention has been directed toward the distinct contribution that nurses can make (10). By virtue of their continuous bedside presence, ICU nurses occupy a particularly strategic position within stewardship activities: their responsibilities encompass monitoring clinical responses to antimicrobial therapy, administering antimicrobial agents, collecting microbiological specimens, identifying adverse drug reactions, and implementing infection prevention and control measures (11, 12).

Despite this recognized potential, nurses’ active participation in antimicrobial stewardship remains constrained in many clinical settings. Multiple barriers have been documented in the existing literature (10, 13), including insufficient stewardship-related knowledge and training, poorly defined professional responsibilities within ASP frameworks, and entrenched hierarchical structures that limit nursing agency (14). In ICU environments specifically, heavy workloads, staffing shortages, and time pressures may further restrict opportunities for meaningful stewardship engagement (15). Collectively, these observations suggest that nurses’ stewardship behaviors are shaped by a complex interplay of individual capabilities, organizational conditions, and motivational factors (16).

The Capability–Opportunity–Motivation–Behavior (COM-B) model provides a widely adopted theoretical framework for understanding behavioral determinants in healthcare. Within this model, behavior is understood as the product of three interacting components: capability (the knowledge and skills required to perform a behavior), opportunity (the external environmental and organizational conditions that enable it), and motivation (the cognitive and affective processes that direct behavioral action) (17–19). The COM-B model has been increasingly applied in implementation science to identify barriers and facilitators to clinical practice and to inform the design of targeted behavioral interventions (20). The closely related Theoretical Domains Framework (TDF) similarly conceptualizes healthcare professional behavior as determined by multiple interacting domains (21). Together, these frameworks offer a structured approach to examining the factors that shape ICU nurses’ participation in antimicrobial stewardship.

In addition to the structural determinants captured by the COM-B model, psychological resources may further influence nurses’ engagement in stewardship-related practice. Nursing professional self-efficacy (NPSE)—defined as an individual’s belief in their capacity to perform occupational tasks effectively and manage workplace demands—represents one such resource (22). Higher NPSE has been associated with greater confidence in clinical decision-making, stronger interdisciplinary collaboration, and more effective communication within healthcare teams (23, 24). In the complex and high-stakes ICU environment, professional self-efficacy may therefore influence a nurse’s willingness to question potentially inappropriate prescriptions, raise antimicrobial-related concerns, and actively participate in multidisciplinary decision-making.

Despite growing recognition of nurses’ potential role in antimicrobial stewardship, empirical studies examining ICU nurses’ stewardship behaviors and their determinants remain limited. The majority of ASP research has focused on physicians and pharmacists (25, 26), while nursing-focused studies have predominantly examined knowledge and attitudes rather than actual stewardship behaviors (27). Moreover, relatively few studies have applied behavioral theory frameworks to systematically investigate the factors influencing nursing participation in ASP activities (28). To address these gaps, this study examined the current status of antimicrobial stewardship participation among ICU nurses and its associated determinants, using the COM-B model as the primary theoretical framework and incorporating NPSE as an additional explanatory variable. By situating the findings within the context of Chinese ICUs, this study aims to contribute evidence that may inform the development of targeted, multilevel strategies to strengthen nursing involvement in antimicrobial stewardship.

## 2 Materials and methods

### 2.1 Study design and participants

This cross-sectional study was conducted in March 2026 across 11 hospitals, including Tangdu Hospital, Beijing Ditan Hospital, the First Affiliated Hospital of Xi’an Jiaotong University, Xiangya Hospital of Central South University, Southwest Hospital of Army Medical University, Hanzhong Central Hospital, Yan’an Central Hospital, Ankang Central Hospital, Yulin Central Hospital, Shaanxi Provincial People’s Hospital, and Xijing Hospital. Eligible participants were ICU nurses who were working in intensive care units during the study period and voluntarily completed the online questionnaire. Questionnaires were excluded if participants refused to participate or did not consent to the use of their data for research, or if key information was missing or clearly unreasonable. The study was approved by the Institutional Ethics Committee of Tangdu Hospital, Fourth Military Medical University (No. K202603-09). Electronic informed consent was obtained from all participants through an online checkbox indicating their agreement to participate and to allow their data to be used for research purposes.

### 2.2 Instrument development

A self-designed questionnaire was developed based on the COM-B framework, with reference to previous studies on nurses’ antimicrobial stewardship engagement and questionnaire-based research on implementation determinants (13, 16, 21, 29). The initial draft was reviewed by 3 experts, who recommended several revisions, including reducing the number of items, to improve content relevance and logical flow. A pilot survey yielded 74 valid questionnaires, and the overall Cronbach’s *α* coefficient was 0.9785. In the formal survey, 532 questionnaires were distributed, and 497 valid questionnaires were included in the final analysis. The overall Cronbach’s *α* coefficient of the final questionnaire was 0.9869. The Cronbach’s α coefficients for the capability, opportunity, motivation, behavior, and NPSE dimensions were 0.9448, 0.9755, 0.9721, 0.9636, and 0.9269, respectively. The KMO value was 0.977, and Bartlett’s test of sphericity was statistically significant (*p* < 0.001).

The questionnaire was administered in Chinese and consisted of 85 closed-ended items and one open-ended question. The closed-ended items were organized into three sections: demographic characteristics, the ICU Nurses’ Participation in ASP Behavior and Influencing Factors (ASP-BF) Scale, and the NPSE Scale.

The demographic section included 15 items on sex, age, highest educational attainment, ICU specialty nurse certification, ASP-related training experience and cumulative training duration, years of nursing practice, years of ICU practice, professional title, job position, nurse level, employment type, ICU type, hospital grade, bed-to-nurse ratio, and the presence of ASP-related policies in the department.

The second section comprised the ICU Nurses’ Participation in ASP-BF Scale, which contained 63 items across four domains: capability (13 items), opportunity (11 items), motivation (23 items), and behavior (16 items). The capability domain included knowledge (C1-C4, 4 items), skills (C5-C9, 5 items), memory, attention, and decision-making (C10-C11, 2 items), and behavioral regulation (C12-C13, 2 items). The opportunity domain included environmental context and resources (O1-O6, 6 items) and social influences (O7-O11, 5 items). The motivation domain included professional role and identity (M1-M2, 2 items), beliefs about capabilities (M3-M5, 3 items), optimism (M6-M8, 3 items), beliefs about consequences (M9-M11, 3 items), reinforcement (M12-M13, 2 items), intentions (M14-M17, 4 items), goals (M18-M19, 2 items), and emotions (M20-M23, 4 items). The behavior domain consisted of 16 items (B1-B16). One open-ended item was added at the end of this section.

The third section was the NPSE Scale (30), which consisted of 7 items across two dimensions: care delivery (N1-N4, 4 items) and professionalism (N5-N7, 3 items). Items in the second and third sections were scored on 5-point scales. For the knowledge items (C1-C4), responses were scored from 5 to 1. All other items were scored from 1 to 5, with reverse scoring applied to M11, M20, and M22. Higher scores indicated higher levels of capability, opportunity, motivation, ASP participation behavior, and NPSE.

### 2.3 Data collection

A combination of convenience and snowball sampling was used for participant recruitment. The questionnaire was administered online through Wenjuanxing, a widely used Chinese survey platform, and was distributed via a WeChat-based link or mini-program. During the study period, the research team initially shared the questionnaire with ICU nurses within their professional networks across the participating hospitals (convenience sampling). These initial participants and nursing colleagues known to the research team were then invited to forward the questionnaire to other eligible ICU nurses, including through workplace communication groups and professional WeChat groups (snowball sampling). Potential participants were informed of the study purpose and the voluntary nature of participation before accessing the questionnaire, and all questionnaires were completed anonymously.

After data collection, responses were screened for quality before analysis. Questionnaires were excluded if the completion time was less than 2 min or if the responses showed obvious logical inconsistencies, including inconsistency between antimicrobial stewardship training and cumulative training duration, an implausible difference between age and years of nursing experience, or ICU working years exceeding total nursing working years. Questionnaires collected during the pilot phase were not included in the final analysis.

### 2.4 Sample size calculation

The required sample size for this cross-sectional study was calculated using the standard equation for population surveys (31):

$$n=\frac{Z^{2}\cdot p\cdot q}{d^{2}}$$

Where Z represents the Z-score corresponding to the 95% confidence interval (1.96), p represents the expected proportion set to 0.5 to achieve maximum sample size when population proportion is unknown, q equals 1-*p* (0.5), and d represents the margin of error set at 5% (0.05). Substituting these values yielded n = (1.96) ^2^ × 0.5 × 0.5 / (0.05) ^2^ = 3.8416 × 0.25/0.0025 = 384. To allow for invalid or incomplete questionnaires, an additional 20% was added, resulting in a target sample size of approximately 460 participants. A total of 497 valid questionnaires were ultimately included, exceeding the minimum required sample size.

### 2.5 Statistical analyses

Data analysis was performed using Stata version 17.0 (StataCorp, College Station, TX, United States) and Mplus version 7.0 (Muthén & Muthén, Los Angeles, CA, USA). Continuous variables were tested for normality and are presented as mean ± standard deviation (SD) when normally distributed or as median and interquartile range (IQR) when non-normally distributed. Categorical variables are presented as number and percentage [n (%)]. For comparisons between two groups, the independent-samples t test was used for normally distributed data, whereas the Wilcoxon rank-sum test was applied for non-normally distributed data. For comparisons among three or more groups, one-way analysis of variance was used for normally distributed variables with homogeneity of variance, while the Kruskal–Wallis test was used for non-normally distributed variables. Item-level distributions of the ICU Nurses’ ASP-BF Scale and the NPSE Scale were summarized descriptively. Pearson correlation analysis was conducted to examine the associations among capability, opportunity, motivation, behavior, and NPSE. For logistic regression analysis, behavior was dichotomized using 70% of the theoretical maximum score as the cut-off value, with scores of ≤70% classified as poor behavior and scores of >70% classified as good behavior (32). Variables with statistical significance in univariate analysis were entered into the multivariate logistic regression model. Odds ratios (ORs) and 95% confidence intervals (CIs) were calculated. Confirmatory factor analysis (CFA) was conducted to examine the factor structure of the ASP-BF Scale. Model fit was evaluated using the root mean square error of approximation (RMSEA), standardized root mean square residual (SRMR), Tucker–Lewis index (TLI), and comparative fit index (CFI). Structural equation modeling (SEM) was further performed to test the hypothesized relationships among capability, opportunity, motivation, behavior, and NPSE, and indirect effects were estimated. In addition, latent profile analysis (LPA) was used to identify potential subgroups of participants. The optimal number of latent profiles was determined by comprehensively considering the Akaike information criterion (AIC), Bayesian information criterion (BIC), sample-size adjusted Bayesian information criterion (aBIC), entropy, Lo–Mendell–Rubin likelihood ratio test (LMR), and bootstrap likelihood ratio test (BLRT). All statistical tests were two-sided, and a *p* value of less than 0.05 was considered statistically significant.

## 3 Results

A total of 532 ICU nurses were recruited for the survey. After excluding 15 nurses who declined to complete the questionnaire, 517 questionnaires remained for screening. During data cleaning, 22 questionnaires were excluded, including 2 with a completion time of less than 120 s, 16 with inconsistent responses to antimicrobial stewardship training and cumulative training duration, 2 with an age–years of nursing experience difference of less than 18 years, and 2 with ICU working years greater than total nursing working years. Ultimately, 497 questionnaires were included in the final analysis.

### 3.1 Participant characteristics

The final analysis included 497 ICU nurses. The mean age was 33.28 ± 6.21 years. Most participants were female (88.13%), had a bachelor’s degree or above (94.16%), worked in tertiary Grade A hospitals (88.13%), and had received antimicrobial stewardship (ASP) training (77.26%). In addition, 36.02% had ICU specialty nurse certification. The mean years of nursing experience and ICU experience were 10.33 ± 6.55 and 5.80 ± 5.29 years, respectively. Detailed participant characteristics and scale scores are shown in Table 1.

**Table 1:** Demographic characteristics.

*N* = 497	*N* (%)	ASP-BF scale								NPSE	
		Capability		Opportunity		Motivation		Behavior			
		Med [q25, q75]	*p*	Mean±SD	*p*	Med [q25, q75]	*p*	Mean±SD	*p*	Mean±SD	*p*
Total score		48.80 ± 9.86		38.42 ± 10.60		82.55 ± 16.86		59.94 ± 13.57		29.60 ± 4.45	
Sex			0.436^c^		0.306^a^		0.540^c^		0.641^a^		0.565^a^
Male	59 (11.87)	48 [43,61]		39.74 ± 11.39		83 [69,100]		60.71 ± 13.81		29.91 ± 4.69	
Female	438 (88.13)	49 [41,55]		38.23 ± 10.49		83 [69,94]		59.83 ± 13.54		29.55 ± 4.42	
Age, years	33.28 ± 6.21										
Highest educational level			0.001^c^		0.008^a^		0.022^c^		0.017^a^		0.074^a^
Junior college or below	29 (5.84)	54 [48,65]		43.48 ± 11.73		89 [81,105]		65.79 ± 14.52		31.03 ± 4.34	
Bachelor’s degree or above	468 (94.16)	48 [41,55]		38.10 ± 10.46		82 [69,93]		59.57 ± 13.43		29.51 ± 4.44	
ICU specialty nurse certification			0.001^c^		0.324^a^		0.091^c^		0.302^a^		0.005^a^
Yes	179 (36.02)	50 [44,57]		39.04 ± 10.34		84 [71,98]		60.77 ± 13.41		30.34 ± 3.72	
No	318 (63.98)	47.5 [40,54]		38.06 ± 10.74		82 [69,91]		59.46 ± 13.65		29.18 ± 4.76	
Antimicrobial stewardship training			<0.001^c^		<0.001^a^		<0.001^c^		<0.001^a^		<0.001^a^
Yes	384 (77.26)	50 [44,57]		39.90 ± 10.13		84 [71,96.5]		61.69 ± 12.58		30.00 ± 4.08	
No	113 (22.74)	42 [37,51]		33.38 ± 10.63		73 [68,87]		53.98 ± 15.07		28.23 ± 5.30	
Cumulative duration of antimicrobial stewardship training			<0.001^c^		0.095^b^		<0.001^c^		0.220^b^		0.013^b^
<8 h	247 (49.70)	44 [39,52]		35.62 ± 10.73		76 [68,89]		56.70 ± 13.80		28.70 ± 4.64	
8–16 h	142 (28.57)	49.5 [44,55]		39.07 ± 9.79		84 [75,96]		60.54 ± 12.27		30.08 ± 3.91	
17–40 h	39 (7.85)	53 [48,58]		41.25 ± 8.19		83 [72,90]		62.48 ± 12.08		30.51 ± 3.28	
>40 h	69 (13.88)	56 [52,65]		45.46 ± 9.11		94 [84,103]		68.79 ± 11.74		31.31 ± 4.67	
Years of nursing experience	10.33 ± 6.55										
Years of ICU experience	5.80 ± 5.29										
Professional title			0.090^c^		0.886^b^		0.023^c^		0.032^b^		0.122^b^
Nurse	79 (15.90)	51 [43,58]		41.68 ± 10.01		85 [73,100]		63.08 ± 12.24		29.34 ± 4.68	
Senior nurse	201 (40.44)	50 [41,58]		39.78 ± 10.41		84 [69,97]		59.93 ± 14.82		29.92 ± 4.09	
Supervisor nurse or above	217 (43.66)	48 [42,53]		35.96 ± 10.48		81 [69,91]		58.79 ± 12.65		29.40 ± 4.67	
Position			0.193^c^		0.619^b^		0.055^c^		0.112^b^		0.245^b^
Staff nurse	388 (78.07)	49 [41,56]		39.07 ± 10.65		84 [70,96.5]		60.04 ± 14.00		29.69 ± 4.47	
Team leader	75 (15.09)	47 [43,52]		35.57 ± 9.78		79 [69,88]		58.89 ± 12.31		28.89 ± 4.62	
Head nurse/nurse manager	34 (6.84)	53 [45,56]		37.23 ± 10.95		83 [70,94]		60.97 ± 11.11		30.14 ± 3.60	
Nursing level			0.170^c^		0.837^b^		0.209^c^		0.205^b^		<0.001^b^
N0	23 (4.63)	53 [43,61]		43.86 ± 10.57		86 [82,98]		62.86 ± 12.82		28.39 ± 6.43	
N1	112 (22.54)	50.5 [41.5,57]		40.57 ± 10.17		84 [71,97.5]		61.63 ± 13.30		29.79 ± 3.90	
N2	223 (44.87)	49 [41,57]		38.70 ± 10.74		84 [69,94]		59.87 ± 14.36		29.82 ± 4.25	
N3	114 (22.94)	47 [41,53]		35.43 ± 10.12		80 [69,92]		57.89 ± 12.91		29.19 ± 5.09	
N4	25 (5.03)	52 [45,53]		34.76 ± 9.17		83 [70,86]		59.52 ± 9.98		29.76 ± 2.93	
Employment type			0.103^c^		0.295^b^		0.541^c^		0.100^b^		0.005^b^
Permanent staff	54 (10.87)	53 [44,58]		38.75 ± 10.25		82.5 [70,96]		61.12 ± 11.70		30.14 ± 3.68	
Contract staff	415 (83.50)	48 [42,55]		38.31 ± 10.50		82 [69,93]		59.76 ± 13.59		29.60 ± 4.40	
Temporary staff	28 (5.63)	45.5 [36.5,56.5]		39.25 ± 12.85		85.5 [70.5,104]		60.17 ± 16.61		28.42 ± 6.19	
ICU type			0.071^c^		0.725^b^		0.031^c^		0.295^b^		0.166^b^
General ICU	133 (26.76)	48 [41,57]		38.22 ± 10.65		81 [69,92]		58.63 ± 14.43		29.45 ± 4.48	
Cardiac ICU	12 (2.41)	52.5 [47,58.5]		42.41 ± 10.05		90 [84.5,97.5]		65.25 ± 8.72		29.75 ± 2.56	
Respiratory ICU	117 (23.54)	48 [41,56]		37.70 ± 10.83		79 [69,95]		60.39 ± 12.59		29.58 ± 4.41	
Neurological ICU	42 (8.45)	47 [41,55]		36.04 ± 11.73		76.5 [69,96]		59 ± 14.53		29.5 ± 5.32	
Emergency ICU	23 (4.63)	54 [50,61]		44.34 ± 11.06		90 [81,108]		65.95 ± 12.58		30.39 ± 4.26	
Other	170 (34.21)	49 [41,54]		38.56 ± 9.85		84 [71,92]		59.68 ± 13.52		29.63 ± 4.39	
Hospital level			<0.001^c^		<0.001^a^		<0.001^c^		0.001^a^		0.102^a^
Tertiary Grade A hospital	438 (88.13)	48 [41,55]		37.74 ± 10.33		82 [69,92]		59.21 ± 13.37		29.48 ± 4.45	
Other	59 (11.87)	54 [45,61]		43.40 ± 11.30		91 [73,107]		65.33 ± 13.85		30.49 ± 4.18	
Bed-to-nurse ratio in the unit			0.659^c^		0.270^b^		0.447^c^		0.660^b^		0.709^b^
1:2.5–3	352 (70.82)	49 [41,55]		37.85 ± 10.80		82 [69,93]		59.41 ± 13.81		29.57 ± 4.52	
1:3–3.5	92 (18.51)	50 [42,57]		40.52 ± 9.43		85 [72,97.5]		61.63 ± 12.96		29.55 ± 4.22	
1:>3.5	53 (10.66)	48 [42,57]		38.52 ± 10.87		81 [69,99]		60.49 ± 12.91		29.86 ± 4.43	
Availability of ASP-related policies in the unit			<0.001^c^		0.343^b^		<0.001^c^		0.009^b^		0.001^b^
Yes	409 (82.29)	51 [43,57]		40.22 ± 9.87		85 [72,98]		61.62 ± 13.03		29.89 ± 4.37	
No	22 (4.43)	44 [36,50]		28.63 ± 11.79		74 [57,89]		49.40 ± 18.51		28.04 ± 6.79	
Unknown	66 (13.28)	40.5 [36,44]		30.46 ± 9.17		69.5 [65,79]		52.98 ± 11.10		28.31 ± 3.67	

ASP-BF scale, ICU Nurses’ participation in antimicrobial stewardship behavior and influencing factors scale; NPSE, Nursing professional self-efficacy. ^a^*t* test; ^b^One-way analysis of variance (ANOVA); ^c^Nonparametric test.

As shown in Table 1, nurses who had received ASP training had significantly higher capability, opportunity, motivation, behavior, and NPSE scores than those without such training (all *p* < 0.001). Longer cumulative ASP training duration was also associated with higher capability, motivation, and NPSE scores (all *p* < 0.05). ICU specialty nurse certification was associated with higher capability and NPSE scores (*p* = 0.001 and *p* = 0.005, respectively). Educational level was significantly associated with capability, opportunity, motivation, and behavior scores (all *p* < 0.05).

Professional title was associated with motivation and behavior scores, whereas nursing level and employment type were associated only with NPSE scores. ICU type was associated with motivation scores, and hospital level was associated with capability, opportunity, motivation, and behavior scores (all *p* ≤ 0.001). In addition, the availability of ASP-related policies in the unit was associated with capability, motivation, behavior, and NPSE scores. No significant differences were observed by sex, position, or bed-to-nurse ratio (all *p* > 0.05).

### 3.2 Distribution of the ASP-BF scale and NPSE scale

The item-level distributions of the ASP-BF Scale and the NPSE Scale are presented in Supplementary Tables S1, S2.

For capability, most participants reported being very or moderately knowledgeable about commonly used antimicrobial agents in the ICU (73.24% for item C2) and about the causes of antimicrobial resistance and its impact on patients (65.80% for item C4). For opportunity, positive responses were also common for support-related items, including head nurse encouragement (58.76% for item O9), multidisciplinary collaboration mechanisms (58.56% for item O10), and timely support from pharmacists or infection control staff (57.34% for item O11). For behavior, the highest proportions of “often” or “always” responses were observed for infection control practices, including standard precautions (76.25% for item B14), hand hygiene (81.29% for item B15), and isolation measures for patients with multidrug-resistant organisms (80.48% for item B16). These findings suggest that infection control-related behaviors were more frequently reported than some other ASP-related behaviors (Supplementary Table S1).

Responses to the NPSE Scale were generally positive. Across all seven items, the majority of participants selected “agree” or “completely agree.” The highest proportion of “completely agree” responses was observed for protecting patients’ privacy and confidentiality (55.33% for item N4), whereas relatively lower proportions were observed for participation in nursing research (34.00% for item N5) and collaboration with nursing associations and professional representatives (34.61% for item N6; Supplementary Table S2).

In response to the open-ended question regarding the main barriers to nurses’ participation in antimicrobial stewardship, many participants reported no obvious barriers or did not provide a specific response. Among the reported barriers, the most commonly mentioned were insufficient professional knowledge and inadequate training in antimicrobial stewardship, limited time due to heavy workload, and staffing shortages. Participants also referred to restricted authority in antimicrobial management, unclear professional roles, and difficulties in communication or recognition from physicians. In addition, several respondents mentioned limited institutional support, insufficient opportunities for learning and participation, and concerns about patient trust.

### 3.3 Correlation analysis

As shown in Supplementary Table S3, capability, opportunity, motivation, behavior, and NPSE were all positively correlated with each other (all *p* < 0.001). Behavior was most strongly correlated with motivation (*r* = 0.80), followed by capability (*r* = 0.75), opportunity (*r* = 0.74), and NPSE (*r* = 0.57). In addition, motivation was positively correlated with capability (*r* = 0.75), opportunity (*r* = 0.81), and NPSE (*r* = 0.59).

### 3.4 Univariate and multivariate analyses

For logistic regression analysis, behavior was dichotomized using 70% of the theoretical maximum score as the cut-off value. A behavior score of ≤70% was classified as poor behavior (*n* = 207, 41.65%), and a score of >70% was classified as good behavior (*n* = 290, 58.35%).

In the univariate logistic regression analysis, capability, opportunity, motivation, NPSE, lack of antimicrobial stewardship training, years of ICU experience, professional title, ICU type, hospital level, and availability of ASP-related policies in the unit were associated with behavior.

In the multivariate logistic regression analysis, capability (OR = 1.07, 95% CI: 1.02–1.12, *p* = 0.006), opportunity (OR = 1.06, 95% CI: 1.01–1.12, *p* = 0.015), motivation (OR = 1.10, 95% CI: 1.06–1.14, *p* < 0.001), and NPSE (OR = 1.10, 95% CI: 1.01–1.19, *p* = 0.022) remained positively associated with behavior, whereas lack of antimicrobial stewardship training was negatively associated with behavior (OR = 0.41, 95% CI: 0.20–0.86, *p* = 0.019; Supplementary Table S4).

### 3.5 Structural equation modeling

The structural equation model is shown in Figure 1, and the corresponding path coefficients are presented in Tables 2, 3. Capability (*β* = 0.41, 95% CI: 0.28–0.53, *p* < 0.001), opportunity (*β* = 0.84, 95% CI: 0.72–0.95, *p* < 0.001), and NPSE (*β* = 0.77, 95% CI: 0.58–0.95, *p* < 0.001) were positively associated with motivation. Motivation was positively associated with behavior (*β* = 0.75, 95% CI: 0.69–0.79, *p* < 0.001).

**Figure 1:**
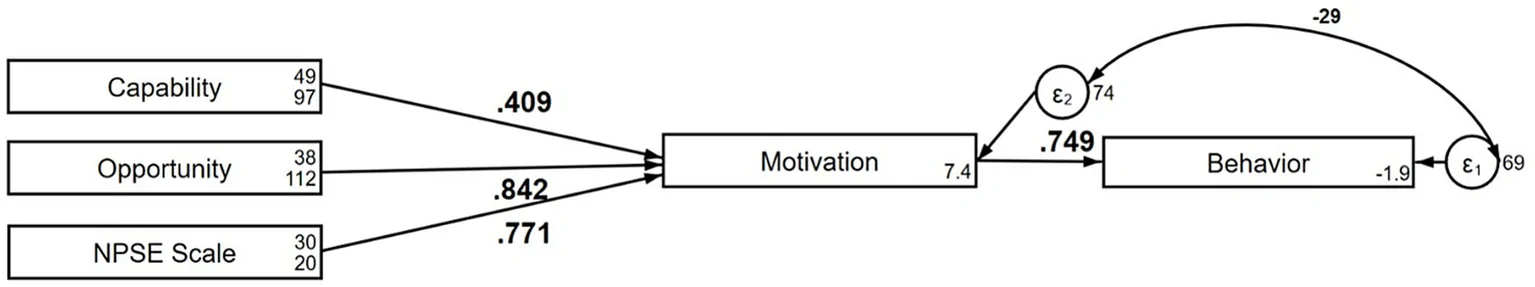
Structural equation modeling. NPSE, Nursing professional self-efficacy.

**Table 2:** Structural equation model path coefficients.

Model paths		Estimate	SE	*z*	*p* > |z|	95% CI
Motivation						
	Capability	0.41	0.06	6.34	<0.001	(0.28,0.53)
	Opportunity	0.84	0.06	14.22	<0.001	(0.72,0.95)
	NPSE	0.77	0.09	8.17	<0.001	(0.58,0.95)
Behavior						
	Motivation	0.75	0.03	29.01	<0.001	(0.69,0.79)

NPSE, Nursing Professional Self-Efficacy.

**Table 3:** Total, direct, and indirect effects in the structural equation model.

Model paths		Total effects		Direct effect		Indirect effect	
		*β* (95% CI)	*p*	*β* (95% CI)	*p*	*β* (95% CI)	*p*
Motivation							
	Capability	0.41 (0.28,0.53)	<0.001	0.41 (0.28,0.53)	<0.001		
	Opportunity	0.84 (0.72,0.95)	<0.001	0.84 (0.72,0.95)	<0.001		
	NPSE	0.77 (0.58,0.95)	<0.001	0.77 (0.58,0.95)	<0.001		
Behavior							
	Motivation	0.75 (0.69,0.79)	<0.001	0.75 (0.69,0.79)	<0.001		
	Capability	0.30 (0.20,0.40)	<0.001			0.30 (0.20,0.40)	<0.001
	Opportunity	0.63 (0.54,0.71)	<0.001			0.63 (0.54,0.71)	<0.001
	NPSE	0.57 (0.43,0.71)	<0.001			0.57 (0.43,0.71)	<0.001

NPSE, Nursing Professional Self-Efficacy.

Indirect effect analysis showed that capability, opportunity, and NPSE were all associated with behavior through motivation. The indirect effects were 0.30 (95% CI: 0.20–0.40, *p* < 0.001) for capability, 0.63 (95% CI: 0.54–0.71, *p* < 0.001) for opportunity, and 0.57 (95% CI: 0.43–0.71, *p* < 0.001) for NPSE. Model fit indices are shown in Supplementary Table S5.

### 3.6 Confirmatory factor analysis

The confirmatory factor analysis supported the factor structure of the ASP-BF Scale (Supplementary Figure S1). The model fit indices were RMSEA = 0.063, SRMR = 0.055, TLI = 0.897, and CFI = 0.891 (Supplementary Table S6). In addition, all factor loadings were statistically significant (all *p* < 0.001; Supplementary Table S7).

### 3.7 Latent profile analysis

The latent profile analysis results are shown in Figure 2 and Supplementary Tables S8, S9. Although the AIC, BIC, and aBIC values continued to decrease as the number of profiles increased, the five-profile model had a non-significant LMR test (*p* = 0.2313). Therefore, the three-profile solution was considered more appropriate, with an entropy of 0.878 and significant LMR and BLRT results.

**Figure 2:**
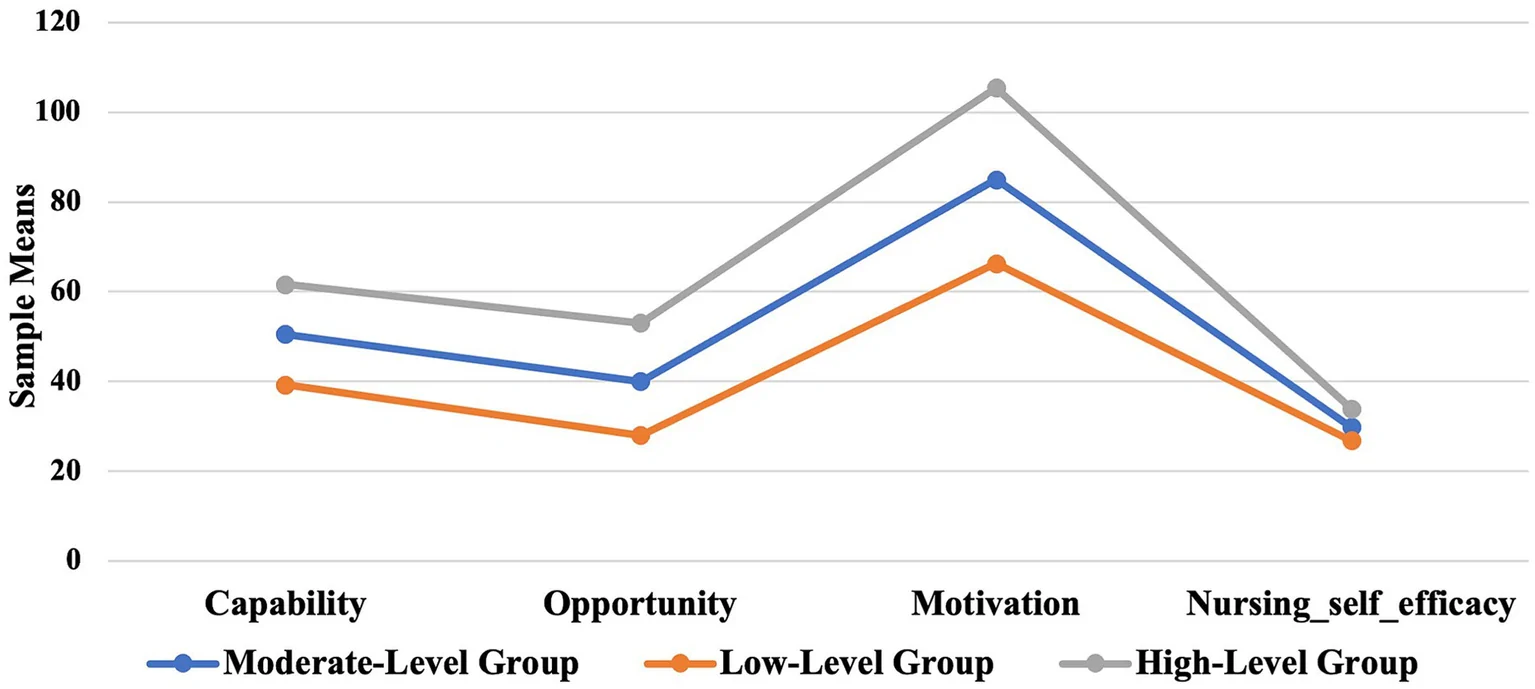
Three-profile solution from latent profile analysis. NPSE, Nursing professional self-efficacy.

In the three-profile model, 178 participants (35.82%) were classified into the low-level group, 219 (44.06%) into the moderate-level group, and 100 (20.12%) into the high-level group. Behavior scores differed significantly among the three profiles (*F* = 337.11, *p* < 0.001; Supplementary Table S9). As shown in Figure 2, the three profiles exhibited a gradient pattern across capability, opportunity, motivation, and nursing self-efficacy.

## 4 Discussion

This cross-sectional study examined the current status and determinants of antimicrobial stewardship participation among ICU nurses, using the COM-B model as a guiding theoretical framework and incorporating NPSE as an additional explanatory variable. Overall, ICU nurses demonstrated generally favorable levels of capability, opportunity, motivation, behavior, and NPSE. Motivation emerged as the factor most directly associated with ASP participation behavior, while capability, opportunity, and NPSE each exerted indirect effects mediated through motivation. ASP training was additionally identified as an important modifiable factor. These findings point to the need for multilevel interventions targeting institutional support, multidisciplinary collaboration and workflow conditions, and structured training to promote nurses’ participation in antimicrobial stewardship.

Item-level analysis revealed that ASP participation was not evenly distributed across activity domains. Infection control–related behaviors—including hand hygiene, standard precautions, and isolation measures for multidrug-resistant organisms—received consistently higher ratings, reflecting relatively active engagement in practices closely embedded in routine bedside care. By contrast, behaviors involving antimicrobial assessment, decision support, and multidisciplinary discussion were less frequently reported. This distribution is consistent with patterns observed in the broader literature, which indicate that nurses tend to be more involved in stewardship tasks integrated into daily nursing workflows—such as infection prevention, medication administration, monitoring, and specimen collection—than in prescribing-or decision-oriented activities (33, 34). Qualitative evidence has similarly shown that nurses respond more favorably to activities such as proper culture technique and intravenous-to-oral antimicrobial switching, whereas tasks perceived as peripheral to routine nursing responsibilities or requiring an expanded professional role are received less positively (35). In ICU settings, nurses have reported limited inclusion in antimicrobial discussions and low confidence in questioning prescriptions or contributing to de-escalation decisions, further reflecting this pattern (33, 36). This may reflect the extent to which infection control practices are already embedded within established ICU nursing roles and workflow priorities, whereas antimicrobial assessment and decision-support activities require a higher degree of interprofessional recognition, structured communication, and organizational provision of participation opportunities (33, 36).

Motivation showed the strongest direct association with ASP participation behavior among the COM-B components examined. This is theoretically consistent with the COM-B model, in which motivation functions as a proximal driver of behavior (37). Within the TDF that underpins COM-B, the motivation construct encompasses multiple domains directly relevant to ASP participation, including social and professional role and identity, beliefs about capabilities, optimism, beliefs about consequences, intentions, goals, reinforcement, and emotional responses (38). Motivation in this context should therefore not be interpreted as simple willingness, but as a composite of psychological processes that collectively determine whether nurses regard stewardship as part of their professional role, feel sufficiently confident to act, anticipate meaningful impact from their actions, form intentions to engage, and maintain the emotional readiness to do so. Existing evidence from ASP research corroborates the relevance of several motivation-related domains. For instance, social and professional role identity and beliefs about consequences have been identified as significant behavioral influences, with skills and behavioral regulation emerging as the strongest overall predictors in at least one COM-B–based study of nursing ASP behavior (17). Qualitative investigation of ASP education implementation has further highlighted that limited conviction about the severity of AMR, insufficient buy-in, and a perceived gap between available training and real-world practice demands represent key motivational barriers (39). These observations suggest that improving nurses’ stewardship participation will require more than enhancing knowledge alone; interventions will also need to address role recognition, confidence in the ASP context, perceived relevance of stewardship activities, and broader psychological engagement, so that nurses are not merely informed but genuinely motivated to participate (17, 37, 39).

Both capability and opportunity were positively associated with motivation and exerted indirect effects on ASP participation behavior. This pattern aligns with the COM-B model’s conceptualization of capability and opportunity as foundational preconditions for behavior that can also modulate motivational processes (37). In the present context, capability encompasses the knowledge, clinical skills, judgment, and behavioral regulation required for active ASP engagement, while opportunity encompasses the external conditions that enable participation—including adequate time, staffing, access to resources, institutional support, workflow structures, and multidisciplinary collaboration arrangements (38). The open-ended responses in this study support this interpretation directly: knowledge deficits, insufficient training, time constraints, staffing shortages, and limited institutional support were among the most frequently cited barriers. These findings are consistent with prior research. Studies using COM-B–based instruments have demonstrated that nurses who had received ASP training scored higher not only on knowledge and skills, but also on measures of environmental context, resources, and social influences, indicating that individual competence and organizational conditions act in concert to shape ASP behavior (17). In ICU settings specifically, lack of educational preparation, limited protected learning time, exclusion from antimicrobial discussions, excessive workload, and inadequate management support have each been documented as significant barriers to nursing engagement (36). Qualitative research has further underscored that even nurses who are motivated to contribute may remain constrained by unclear role boundaries, hierarchical team dynamics, workload pressures, and insufficient organizational structures (33, 39). Considered together, these findings highlight that translating nurses’ willingness to engage into sustained ASP practice depends critically on the concurrent presence of adequate individual capability and a clinical environment that affords genuine opportunity.

NPSE was positively associated with motivation and exerted an indirect effect on ASP participation behavior, suggesting that professional self-efficacy constitutes a substantive rather than peripheral determinant of stewardship engagement. In practical terms, nurses with higher professional self-efficacy may be better positioned to communicate concerns about antimicrobial prescribing, proactively monitor treatment responses, and participate in stewardship-related activities within everyday ICU care. The existing literature offers converging evidence on the role of confidence in shaping nursing involvement in stewardship. Multicentre survey data have shown that nurses can hold positive perceptions of ASPs and express confidence in performing stewardship activities—including reviewing antimicrobial use, advising on treatment duration, identifying unnecessary treatment, and participating in case discussions—even when overall involvement remains limited (40). A significant positive association has also been reported between nurses’ recognition of their own stewardship-relevant practices and their confidence to carry them out, with safety culture identified as an important organizational constraint on this relationship (41). Lack of self-confidence has been specifically characterized as a modifiable barrier to nurse-led stewardship behavior, while the capacity to assess patients and communicate findings to the clinical team has been described as a key facilitator (42). Review evidence further indicates that nursing contributions to ASP are frequently limited by reluctance to raise concerns, particularly when this involves questioning prescribing decisions or engaging with more senior clinicians (40). Collectively, these findings support the value of targeting professional self-efficacy in future ASP interventions, especially where the goal is to strengthen nurses’ capacity for communication, interdisciplinary collaboration, and sustained engagement in complex ICU environments.

Nurses who had received ASP training demonstrated significantly higher scores across all COM-B domains and NPSE, and absence of training remained independently and negatively associated with ASP participation behavior in the multivariable analysis. Notably, longer cumulative training duration was associated with higher capability, motivation, and NPSE scores, suggesting that the extent of training exposure may matter beyond simply whether training was received or not. These findings position ASP training as one of the most actionable modifiable factors for improving participation. Convergent evidence from COM-B–based surveys shows that trained nurses consistently outperform untrained peers across behavior, capability, opportunity, and motivation scores (17). Educational intervention studies have further demonstrated improvements across multiple dimensions following stewardship-specific training, including knowledge, clinical practice, role awareness, and professional confidence (43, 44). Access to such training in ICU settings nonetheless appears to remain limited, with data suggesting that only a minority of nurses report the availability of formal ASP education and training programs in their units (36). These considerations suggest that future ASP training for nurses should be ongoing, structurally embedded within clinical workflows, and designed to bridge the gap between theoretical knowledge and real-world practice demands, rather than being confined to isolated didactic sessions.

Open-ended responses provided additional qualitative insight into the barriers shaping ASP participation, with four main clusters emerging: insufficient knowledge and training, heavy workload and time constraints, staffing shortages, and limited authority or ill-defined professional roles. This knowledge gap may be particularly pronounced when caring for immunocompromised patients, such as those with HIV/AIDS, whose complex opportunistic infections and antiretroviral-antibiotic interactions demand a level of stewardship-relevant expertise that general ICU training may not adequately address (45). Subsidiary concerns included communication difficulties with physicians, inadequate policy or institutional support, and restricted opportunities for professional development. These qualitative findings closely align with the quantitative results, particularly reinforcing the centrality of capability and opportunity as determinants of ASP engagement. Comparable barriers have been extensively documented in the literature. Reviews of the nursing literature identify limited education, role ambiguity, and reluctance to challenge prescribing decisions within multidisciplinary teams as pervasive constraints (10). Qualitative studies have similarly highlighted organizational barriers, hierarchical team dynamics, collaborative difficulties, and staffing pressures as factors limiting nursing involvement in stewardship (15, 33). The communicative dimension of nursing stewardship participation merits particular attention, as the ability to interact effectively with prescribers and the broader team is central to many nurse-led ASP behaviors (11). These observations emphasize that nurses’ ASP participation is constrained not only by individual capability gaps, but also by the structural legitimacy afforded to the nursing role within stewardship frameworks and the extent to which organizations actively enable that participation.

The present findings carry several practical implications. At the individual level, ASP-related education should be regular, structured, and practice-oriented, with a specific focus on strengthening nurses’ knowledge of antimicrobial management, clinical assessment skills, communication competencies, and confidence in stewardship-related decision support (17, 43, 44). At the team level, concerted effort should be directed toward improving communication and fostering multidisciplinary collaboration among nurses, physicians, pharmacists, and infection control personnel, with the explicit aim of ensuring that nurses are consistently included in antimicrobial discussions and care planning (10, 33, 39). At the organizational level, institutions may need to clarify and formalize nurses’ roles within ASP governance structures, optimize workflow to create protected time for stewardship-related activities, ensure adequate staffing, and reinforce participation through policy development and departmental support mechanisms (36, 41). These implications collectively underscore that meaningful improvement in nurses’ ASP participation is unlikely to result from educational initiatives alone, but instead requires coordinated action spanning individual, team, and organizational levels (37, 38).

This study has several limitations that should be considered when interpreting the findings. First, participants were recruited through a combination of convenience and snowball sampling via an online platform. Because recruitment propagated through existing professional networks, the sample may over-represent nurses who are more connected to professional communities or more interested in antimicrobial stewardship, and the response rate could not be determined; selection bias and limited representativeness therefore cannot be excluded. Generalization of these findings to ICU nurses in other regions or healthcare contexts should be approached with caution. Second, the cross-sectional design precludes causal inference regarding the relationships among capability, opportunity, motivation, NPSE, and ASP participation behavior, notwithstanding the use of structural equation modeling to evaluate hypothesized directional pathways. Third, all data were derived from self-report questionnaires, which are susceptible to recall bias and social desirability effects, particularly for behavior-related items. These concerns were partially mitigated through the application of predefined eligibility criteria, systematic data cleaning, and rigorous psychometric validation, with the questionnaire demonstrating strong internal consistency and acceptable construct validity. Future studies would benefit from employing probability-based or multicentre sampling strategies, longitudinal or prospective designs, and more objective assessments of stewardship behavior to extend and validate the present findings.

In summary, ICU nurses demonstrated generally favorable levels of capability, opportunity, motivation, behavior, and NPSE in relation to antimicrobial stewardship participation. Motivation was the proximal determinant most directly associated with participation behavior, while capability, opportunity, and NPSE operated through motivational pathways. Future interventions should address institutional support structures, multidisciplinary collaboration mechanisms, and workflow conditions alongside the provision of structured, practice-oriented training to enhance and sustain nurses’ participation in antimicrobial stewardship.

## Data Availability

The raw data supporting the conclusions of this article will be made available by the authors, without undue reservation.
