# Supplementary material for: Participation in antimicrobial stewardship among intensive care unit nurses: a cross-sectional study based on the COM-B model
Source: Front Public Health. 2026 Sep 17;14:1922847. doi: 10.3389/fpubh.2026.1922847 (PMC13627333; doi:10.3389/fpubh.2026.1922847)
Supplement: Supplementary file 1 [file Data_Sheet_1.DOCX]

Supplementary Material

# 1 Supplementary Figures and Tables

## 1.1 Supplementary Figures

**Supplementary Figure 1. Confirmatory Factor Analysis Model of the ASP-BF Scale**

**Supplementary Table 1. Distribution of responses to the ICU Nurses’ Participation in Antimicrobial Stewardship Behavior and Influencing Factors Scale**

| **Capability** | | | | | |
| --- | --- | --- | --- | --- | --- |
| **Knowledge** | **Very knowledgeable** | **Moderately knowledgeable** | **Generally knowledgeable** | **Slightly unknowledgeable** | **Very unknowledgeable** |
| C1. I understand the relevant content of antimicrobial stewardship (ASP) in my hospital, including related policies, regulations, and the roles and responsibilities of nurses. | 125 (25.15) | 171 (34.41) | 158 (31.79) | 34 (6.84) | 9 (1.81) |
| C2. I am familiar with the administration timing, routes, and precautions for commonly used antimicrobial agents in the ICU. | 175 (35.21) | 189 (38.03) | 111 (22.33) | 18 (3.62) | 4 (0.80) |
| C3. I know how to make a preliminary judgment about the type and severity of infection based on patients’ symptoms, signs, and test results. | 124 (24.95) | 180 (36.22) | 162 (32.60) | 28 (5.63) | 3 (0.60) |
| C4. I understand the causes of antimicrobial resistance and its impact on patients. | 123 (24.75) | 204 (41.05) | 145 (29.18) | 20 (4.02) | 5 (1.01) |
| **Skills** | **Never** | **Rarely** | **Sometimes** | **Often** | **Always** |
| C5. I am able to collect pathogen specimens in a standardized manner or guide patients to collect specimens correctly. | 13 (2.62) | 19 (3.82) | 61 (12.27) | 205 (41.25) | 199 (40.04) |
| C6. I am able to identify adverse reactions after antimicrobial use and manage them promptly. | 7 (1.41) | 30 (6.04) | 139 (27.97) | 197 (39.64) | 124 (24.95) |
| C7. I am able to administer antimicrobials correctly according to their pharmacological characteristics (time-dependent or concentration-dependent). | 6 (1.21) | 19 (3.82) | 98 (19.72) | 187 (37.63) | 187 (37.63) |
| C8. I am able to accurately assess whether a patient meets the criteria for switching from intravenous to oral antimicrobial therapy. | 18 (3.62) | 81 (16.30) | 156 (31.39) | 121 (24.35) | 121 (24.35) |
| C9. I have mastered the professional skills required to participate in ASP. | 15 (3.02) | 74 (14.89) | 165 (33.20) | 141 (28.37) | 102 (20.52) |
| **Memory, attention, and decision processes** |  |  |  |  |  |
| C10. In the busy ICU work environment, I am able to continuously pay attention to patients’ antimicrobial use. | 10 (2.01) | 43 (8.65) | 157 (31.59) | 165 (33.20) | 122 (24.55) |
| C11. I am able to apply ASP-related knowledge in actual practice. | 7 (1.41) | 46 (9.26) | 169 (34.00) | 154 (30.99) | 121 (24.35) |
| Behavioral regulation |  |  |  |  |  |
| C12. When encountering barriers, I have coping strategies that enable me to continue participating in ASP. | 13 (2.62) | 62 (12.47) | 188 (37.83) | 135 (27.16) | 99 (19.92) |
| C13. I regularly reflect on my performance in ASP and seek ways to improve it. | 11 (2.21) | 63 (12.68) | 175 (35.21) | 141 (28.37) | 107 (21.53) |
| **Opportunity** | | | | | |
| **Environmental context and resources** |  |  |  |  |  |
| O1. The ICU where I work provides sufficient time for nurses to participate in ASP activities. | 24 (4.83) | 89 (17.91) | 180 (36.22) | 120 (24.14) | 84 (16.90) |
| O2. I can conveniently access ASP-related resources, such as guidelines, protocols, and decision-support tools. | 17 (3.42) | 76 (15.29) | 178 (35.81) | 133 (26.76) | 93 (18.71) |
| O3. The organizational culture of the hospital supports nurses’ participation in ASP. | 21 (4.23) | 66 (13.28) | 181 (36.42) | 127 (25.55) | 102 (20.52) |
| O4. The unit has sufficient staffing to enable nurses to participate in ASP activities. | 20 (4.02) | 93 (18.71) | 176 (35.41) | 116 (23.34) | 92 (18.51) |
| O5. The hospital information system effectively supports nurses’ participation in ASP. | 24 (4.83) | 65 (13.08) | 187 (37.63) | 123 (24.75) | 98 (19.72) |
| O6. There are clear mechanisms and procedures in the workflow for nurses to participate in ASP. | 20 (4.02) | 67 (13.48) | 175 (35.21) | 130 (26.16) | 105 (21.13) |
| **Social influences** |  |  |  |  |  |
| O7. My colleagues actively participate in ASP activities. | 15 (3.02) | 72 (14.49) | 155 (31.19) | 152 (30.58) | 103 (20.72) |
| O8. ICU physicians support nurses’ participation in ASP-related decision-making. | 24 (4.83) | 80 (16.10) | 153 (30.78) | 132 (26.56) | 108 (21.73) |
| O9. The head nurse encourages us to participate in ASP. | 10 (2.01) | 42 (8.45) | 153 (30.78) | 155 (31.19) | 137 (27.57) |
| O10. The hospital has a multidisciplinary collaboration mechanism (physicians, nurses, pharmacists, and infection control staff) to support ASP. | 12 (2.41) | 51 (10.26) | 143 (28.77) | 170 (34.21) | 121 (24.35) |
| O11. Pharmacists and infection control staff can provide nurses with timely ASP-related support. | 17 (3.42) | 46 (9.26) | 149 (29.98) | 166 (33.40) | 119 (23.94) |
| **Motivation** | | | | | |
| **Professional role and identity** |  |  |  |  |  |
| M1. Participation in ASP is an important part of the ICU nurse’s professional role. | 11 (2.21) | 41 (8.25) | 147 (29.58) | 159 (31.99) | 139 (27.97) |
| M2. Participation in ASP can enhance my professional value and sense of professional identity. | 11 (2.21) | 33 (6.64) | 147 (29.58) | 175 (35.21) | 131 (26.36) |
| **Beliefs about capabilities** |  |  |  |  |  |
| M3. Even when work is busy or patients are critically ill, I am still confident in participating in ASP. | 11 (2.21) | 51 (10.26) | 170 (34.21) | 145 (29.18) | 120 (24.14) |
| M4. When I believe that an antimicrobial prescription may be inappropriate, I am confident in raising questions with the physician. | 10 (2.01) | 33 (6.64) | 156 (31.39) | 160 (32.19) | 138 (27.77) |
| M5. If I want to participate in ASP, I am capable of doing so. | 9 (1.81) | 43 (8.65) | 172 (34.61) | 153 (30.78) | 120 (24.14) |
| **Optimism** |  |  |  |  |  |
| M6. I believe that nurses’ participation in ASP can positively affect patient outcomes. | 11 (2.21) | 25 (5.03) | 142 (28.57) | 165 (33.20) | 154 (30.99) |
| M7. I am optimistic about the future implementation of ASP in the ICU. | 11 (2.21) | 27 (5.43) | 144 (28.97) | 175 (35.21) | 140 (28.17) |
| M8. I believe that, through continued effort, nurses can play a greater role in ASP. | 9 (1.81) | 23 (4.63) | 139 (27.97) | 175 (35.21) | 151 (30.38) |
| **Beliefs about consequences** |  |  |  |  |  |
| M9. My participation in ASP can improve patient safety and reduce antimicrobial resistance. | 9 (1.81) | 30 (6.04) | 154 (30.99) | 170 (34.21) | 134 (26.96) |
| M10. My ASP participation behaviors can have a positive effect on infection control. | 8 (1.61) | 30 (6.04) | 150 (30.18) | 171 (34.41) | 138 (27.77) |
| M11. If I question an antimicrobial prescription, it may affect my relationship with the physician. | 103 (20.72) | 102 (20.52) | 131 (26.36) | 101 (20.32) | 60 (12.07) |
| **Reinforcement** |  |  |  |  |  |
| M12. When I participate in ASP, I receive recognition from colleagues or supervisors. | 12 (2.41) | 37 (7.44) | 184 (37.02) | 154 (30.99) | 110 (22.13) |
| M13. Participation in ASP brings me positive feedback and a sense of accomplishment. | 11 (2.21) | 34 (6.84) | 176 (35.41) | 155 (31.19) | 121 (24.35) |
| **Intention** |  |  |  |  |  |
| M14. I intend to participate in ASP activities during my next shift. | 15 (3.02) | 61 (12.27) | 163 (32.80) | 145 (29.18) | 113 (22.74) |
| M15. I plan to actively participate in ASP-related work this week. | 19 (3.82) | 66 (13.28) | 182 (36.62) | 128 (25.75) | 102 (20.52) |
| M16. I have a strong willingness to participate in ASP. | 13 (2.62) | 58 (11.67) | 178 (35.81) | 139 (27.97) | 109 (21.93) |
| M17. I intend to continue increasing my level of participation in ASP in the future. | 12 (2.41) | 49 (9.86) | 164 (33.00) | 153 (30.78) | 119 (23.94) |
| **Goals** |  |  |  |  |  |
| M18. ASP is a priority in my daily nursing practice. | 13 (2.62) | 64 (12.88) | 169 (34.00) | 140 (28.17) | 111 (22.33) |
| M19. I have set specific work goals for my participation in ASP. | 17 (3.42) | 88 (17.71) | 192 (38.63) | 119 (23.94) | 81 (16.30) |
| **Emotion** |  |  |  |  |  |
| M20. I feel anxious when questioning an antimicrobial prescription* (reverse scored). | 73 (14.69) | 129 (25.96) | 186 (37.42) | 61 (12.27) | 48 (9.66) |
| M21. I feel confident when participating in discussions about antimicrobial stewardship. | 12 (2.41) | 62 (12.47) | 220 (44.27) | 118 (23.74) | 85 (17.10) |
| M22. Thinking about participating in antimicrobial stewardship makes me feel stressed. | 48 (9.66) | 116 (23.34) | 213 (42.86) | 73 (14.69) | 47 (9.46) |
| M23. Participating in ASP gives me a sense of accomplishment. | 11 (2.21) | 53 (10.66) | 202 (40.64) | 133 (26.76) | 98 (19.72) |
| **Behavior** | | | | | |
| **Assessment and monitoring** |  |  |  |  |  |
| B1. I monitor patients’ therapeutic responses, clinical effectiveness, and adverse reactions after antimicrobial use. | 14 (2.82) | 63 (12.68) | 172 (34.61) | 144 (28.97) | 104 (20.93) |
| B2. I assess whether a patient meets the criteria for switching from intravenous to oral antimicrobial therapy. | 27 (5.43) | 84 (16.90) | 174 (35.01) | 116 (23.34) | 96 (19.32) |
| B3. I pay attention to the duration of antimicrobial therapy and provide timely reminders for review. | 22 (4.43) | 81 (16.30) | 163 (32.80) | 133 (26.76) | 98 (19.72) |
| B4. I supervise the correct timing and route of antimicrobial administration. | 13 (2.62) | 38 (7.65) | 143 (28.77) | 171 (34.41) | 132 (26.56) |
| **Specimen collection** |  |  |  |  |  |
| B5. I collect microbiological specimens in a standardized manner before antimicrobial use. | 13 (2.62) | 31 (6.24) | 126 (25.35) | 159 (31.99) | 168 (33.80) |
| B6. I ensure that the timing and quality of specimen collection meet requirements. | 9 (1.81) | 22 (4.43) | 114 (22.94) | 170 (34.21) | 182 (36.62) |
| B7. I promptly follow up on microbiological culture results. | 14 (2.82) | 36 (7.24) | 145 (29.18) | 163 (32.80) | 139 (27.97) |
| **Communication and collaboration** |  |  |  |  |  |
| B8. When I have concerns about antimicrobial use, I communicate with the physician. | 14 (2.82) | 29 (5.84) | 139 (27.97) | 156 (31.39) | 159 (31.99) |
| B9. I participate in multidisciplinary discussions on antimicrobial use. | 34 (6.84) | 79 (15.90) | 170 (34.21) | 106 (21.33) | 108 (21.73) |
| B10. I consult pharmacists or infection control staff about antimicrobial-related issues. | 16 (3.22) | 71 (14.29) | 178 (35.81) | 112 (22.54) | 120 (24.14) |
| **Patient education** |  |  |  |  |  |
| B11. I educate patients/family members about the correct use of antimicrobials. | 20 (4.02) | 45 (9.05) | 154 (30.99) | 144 (28.97) | 134 (26.96) |
| B12. I explain the risks of antimicrobial resistance to patients/family members. | 21 (4.23) | 63 (12.68) | 156 (31.39) | 134 (26.96) | 123 (24.75) |
| B13. I educate patients/family members on completing the full course of antimicrobial therapy. | 26 (5.23) | 64 (12.88) | 146 (29.38) | 136 (27.36) | 125 (25.15) |
| **Infection control** |  |  |  |  |  |
| B14. I strictly implement standard precautions to prevent healthcare-associated infections. | 10 (2.01) | 22 (4.43) | 86 (17.30) | 143 (28.77) | 236 (47.48) |
| B15. I strictly adhere to hand hygiene protocols to reduce cross-infection. | 7 (1.41) | 15 (3.02) | 71 (14.29) | 115 (23.14) | 289 (58.15) |
| B16. I implement isolation measures for patients with multidrug-resistant organisms. | 4 (0.80) | 15 (3.02) | 78 (15.69) | 109 (21.93) | 291 (58.55) |

**Supplementary Table 2. Distribution of Nursing Professional Self-Efficacy Scale**

| **NPSE Scale** | **Completely disagree** | **Disagree** | **Uncertain** | **Agree** | **Completely agree** |
| --- | --- | --- | --- | --- | --- |
| **Care delivery** |  |  |  |  |  |
| N1. I am able to provide safe and effective care in accordance with evidence-based nursing practice. | 4 (0.80) | 5 (1.01) | 34 (6.84) | 245 (49.30) | 209 (42.05) |
| N2. I am able to promote health and safety at the community and societal levels. | 6 (1.21) | 5 (1.01) | 51 (10.26) | 265 (53.32) | 170 (34.21) |
| N3. I am able to ensure that healthcare delivery consistently meets the highest professional standards. | 5 (1.01) | 1 (0.20) | 44 (8.85) | 257 (51.71) | 190 (38.23) |
| N4. I am able to protect patients’ privacy and confidentiality. | 4 (0.80) | 2 (0.40) | 21 (4.23) | 195 (39.24) | 275 (55.33) |
| **Professionalism** |  |  |  |  |  |
| N5. I am able to participate in nursing research. | 3 (0.60) | 11 (2.21) | 81 (16.3) | 233 (46.88) | 169 (34.00) |
| N6. I am able to collaborate with nursing associations and professional representatives to ensure the best standards of care in practice. | 7 (1.41) | 5 (1.01) | 68 (13.68) | 245 (49.30) | 172 (34.61) |
| N7. I am able to report colleagues’ abuse or unethical behavior to the relevant regulatory authorities. | 10 (2.01) | 13 (2.62) | 70 (14.08) | 223 (44.87) | 181 (36.42) |

**Abbreviations:** NPSE, Nursing Professional Self-Efficacy.

**Supplementary Table 3. Correlation Analysis**

|  | **Capability** | **Opportunity** | **Motivation** | **Behavior** | **NPSE Scale** |
| --- | --- | --- | --- | --- | --- |
| **Capability** | 1 |  |  |  |  |
| **Opportunity** | 0.80 (P＜0.001) | 1 |  |  |  |
| **Motivation** | 0.75 (P＜0.001) | 0.81 (P＜0.001) | 1 |  |  |
| **Behavior** | 0.75 (P＜0.001) | 0.74 (P＜0.001) | 0.80 (P＜0.001) | 1 |  |
| **NPSE Scale** | 0.54 (P＜0.001) | 0.46 (P＜0.001) | 0.59 (P＜0.001) | 0.57 (P＜0.001) | 1 |

**Abbreviations:** NPSE Scale, Nursing Professional Self-Efficacy Scale.

**Supplementary Table 4. Univariate and Multivariate Logistic Regression for Behavior**

| **Behavior** | **Univariate Logistic Regression** | | **Multivariate Logistic Regression** | |
| --- | --- | --- | --- | --- |
|  | OR (95%CI) | P | OR (95%CI) | P |
| **Capability** | 1.22 (1.18,1.26) | <0.001 | 1.07 (1.02,1.12) | 0.006 |
| **Opportunity** | 1.22 (1.17,1.26) | <0.001 | 1.06 (1.01,1.12) | 0.015 |
| **Motivation** | 1.17 (1.13,1.20) | <0.001 | 1.10 (1.06,1.14) | <0.001 |
| **NPSE Scale** | 1.29 (1.22,1.37) | <0.001 | 1.10 (1.01,1.19) | 0.022 |
| **Sex** |  |  |  |  |
| Male | Ref |  |  |  |
| Female | 0.95 (0.54,1.66) | 0.872 |  |  |
| **Age, years** | 0.97 (0.94,1.00) | 0.080 |  |  |
| **Highest educational level** |  |  |  |  |
| Junior college or below | Ref |  |  |  |
| Bachelor’s degree or above | 0.42 (0.17,1.01) | 0.055 |  |  |
| **ICU specialty nurse certification** |  |  |  |  |
| Yes | Ref |  |  |  |
| No | 0.87 (0.60,1.27) | 0.501 |  |  |
| **Antimicrobial stewardship training** |  |  |  |  |
| Yes | Ref |  | Ref |  |
| No | 0.30 (0.19,0.47) | <0.001 | 0.41 (0.20,0.86) | 0.019 |
| **Years of nursing experience** | 0.98 (0.95,1.00) | 0.183 |  |  |
| **Years of ICU experience** | 0.96 (0.93,0.99) | 0.041 | 1.01 (0.95,1.07) | 0.711 |
| **Professional title** |  |  |  |  |
| Nurse | Ref |  | Ref |  |
| Senior nurse | 0.62 (0.35,1.08) | 0.092 | 0.49 (0.21,1.17) | 0.110 |
| Supervisor nurse or above | 0.51 (0.29,0.88) | 0.016 | 0.54 (0.21,1.37) | 0.200 |
| **Position** |  |  |  |  |
| Staff nurse | Ref |  |  |  |
| Team leader | 0.91 (0.55,1.50) | 0.718 |  |  |
| Head nurse / nurse manager | 1.31 (0.63,2.73) | 0.464 |  |  |
| **Nursing level** |  |  |  |  |
| N0 | Ref |  |  |  |
| N1 | 0.78 (0.29,2.07) | 0.629 |  |  |
| N2 | 0.56 (0.22,1.43) | 0.232 |  |  |
| N3 | 0.46 (0.17,1.22) | 0.123 |  |  |
| N4 | 0.92 (0.27,3.15) | 0.907 |  |  |
| **Employment type** |  |  |  |  |
| Permanent staff | Ref |  |  |  |
| Contract staff | 0.95 (0.53,1.69) | 0.868 |  |  |
| Temporary staff | 1.06 (0.41,2.70) | 0.899 |  |  |
| **Work environment** |  |  |  |  |
| **ICU type** |  |  |  |  |
| General ICU | Ref |  | Ref |  |
| Cardiac ICU | 4.10 (0.86,19.4) | 0.075 | 3.77 (0.27,52.5) | 0.324 |
| Respiratory ICU | 1.10 (0.66,1.81) | 0.706 | 1.44 (0.66,3.15) | 0.354 |
| Neurological ICU | 0.82 (0.41,1.64) | 0.580 | 1.06 (0.36,3.15) | 0.907 |
| Emergency ICU | 2.95 (1.03,8.43) | 0.042 | 1.19 (0.17,8.14) | 0.853 |
| Other | 1.20 (0.76,1.90) | 0.429 | 0.92 (0.44,1.93) | 0.843 |
| **Hospital level** |  |  |  |  |
| Tertiary Grade A hospital | Ref |  | Ref |  |
| Other | 2.07 (1.13,3.80) | 0.018 | 1.09 (0.35,3.31) | 0.879 |
| **Bed-to-nurse ratio in the unit** |  |  |  |  |
| 1:2.5–3 | Ref |  |  |  |
| 1:3–3.5 | 1.32 (0.82,2.13) | 0.243 |  |  |
| 1:>3.5 | 0.89 (0.50,1.60) | 0.714 |  |  |
| **Availability of ASP-related policies in the unit** |  |  |  |  |
| Yes | Ref |  | Ref |  |
| No | 0.31 (0.13,0.77) | 0.012 | 0.79 (0.18,3.44) | 0.755 |
| Unknown | 0.22 (0.12,0.39) | <0.001 | 1.64 (0.66,4.05) | 0.283 |

**Abbreviations:** NPSE, Nursing Professional Self-Efficacy.

**Supplementary Table 5. Model Fit** **for the Structural Equation Model**

| **Indicators** | **Reference** | **Results** |
| --- | --- | --- |
| **RMSEA** | <0.08 Good | 0.115 |
| **SRMR** | <0.08 Good | 0.010 |
| **TLI** | >0.8 Good | 0.964 |
| **CFI** | >0.8 Good | 0.990 |

**Supplementary Table 6. Model Fit for the Confirmatory Factor Analysis Model**

| **Indicators** | **Reference** | **Results** |
| --- | --- | --- |
| **RMSEA** | <0.08 Good | 0.063 |
| **SRMR** | <0.08 Good | 0.055 |
| **TLI** | >0.8 Good | 0.897 |
| **CFI** | >0.8 Good | 0.891 |

**Supplementary Table 7. Factor Loadings from Confirmatory Factor Analysis**

|  |  | **Coefficient** | **SD.** | **z** | **P>\|z\|** | **95%CI** |
| --- | --- | --- | --- | --- | --- | --- |
| **C1** | **Capability** | 1 |  |  |  |  |
| **C2** | **Capability** | 0.81 | 0.05 | 16.34 | <0.001 | (0.70,0.90) |
| **C3** | **Capability** | 0.91 | 0.05 | 19.29 | <0.001 | (0.81,1.00) |
| **C4** | **Capability** | 0.85 | 0.05 | 17.05 | <0.001 | (0.75,0.94) |
| **C5** | **Capability** | 0.70 | 0.07 | 9.51 | <0.001 | (0.55,0.84) |
| **C6** | **Capability** | 1.06 | 0.08 | 13.93 | <0.001 | (0.90,1.20) |
| **C7** | **Capability** | 0.94 | 0.07 | 12.71 | <0.001 | (0.79,1.08) |
| **C8** | **Capability** | 1.48 | 0.10 | 15.38 | <0.001 | (1.28,1.66) |
| **C9** | **Capability** | 1.52 | 0.09 | 16.41 | <0.001 | (1.33,1.70) |
| **C10** | **Capability** | 1.44 | 0.09 | 16.48 | <0.001 | (1.26,1.61) |
| **C11** | **Capability** | 1.48 | 0.09 | 16.95 | <0.001 | (1.31,1.65) |
| **C12** | **Capability** | 1.47 | 0.09 | 16.44 | <0.001 | (1.29,1.64) |
| **C13** | **Capability** | 1.47 | 0.09 | 16.38 | <0.001 | (1.29,1.64) |
| **O1** | **Opportunity** | 1.00 |  |  |  |  |
| **O2** | **Opportunity** | 1.00 | 0.04 | 28.48 | <0.001 | (0.93,1.07) |
| **O3** | **Opportunity** | 1.04 | 0.04 | 29.51 | <0.001 | (0.97,1.11) |
| **O4** | **Opportunity** | 1.05 | 0.04 | 28.65 | <0.001 | (0.97,1.11) |
| **O5** | **Opportunity** | 1.07 | 0.03 | 30.78 | <0.001 | (1.00,1.14) |
| **O6** | **Opportunity** | 1.05 | 0.04 | 29.56 | <0.001 | (0.98,1.12) |
| **O7** | **Opportunity** | 1.02 | 0.04 | 29.11 | <0.001 | (0.95,1.09) |
| **O8** | **Opportunity** | 1.08 | 0.04 | 28.47 | <0.001 | (1.00,1.15) |
| **O9** | **Opportunity** | 0.92 | 0.04 | 25.81 | <0.001 | (0.84,0.98) |
| **O10** | **Opportunity** | 0.90 | 0.04 | 24.19 | <0.001 | (0.82,0.96) |
| **O11** | **Opportunity** | 0.94 | 0.04 | 25.32 | <0.001 | (0.86,1.00) |
| **M1** | **Motivation** | 1.00 |  |  |  |  |
| **M2** | **Motivation** | 0.99 | 0.03 | 30.31 | <0.001 | (0.92,1.04) |
| **M3** | **Motivation** | 1.11 | 0.04 | 25.28 | <0.001 | (1.02,1.19) |
| **M4** | **Motivation** | 0.94 | 0.05 | 20.81 | <0.001 | (0.85,1.03) |
| **M5** | **Motivation** | 1.04 | 0.04 | 24 | <0.001 | (0.95,1.12) |
| **M6** | **Motivation** | 1.01 | 0.04 | 23.13 | <0.001 | (0.92,1.09) |
| **M7** | **Motivation** | 0.99 | 0.04 | 22.74 | <0.001 | (0.90,1.07) |
| **M8** | **Motivation** | 0.97 | 0.04 | 23.06 | <0.001 | (0.88,1.05) |
| **M9** | **Motivation** | 0.98 | 0.04 | 22.84 | <0.001 | (0.89,1.06) |
| **M10** | **Motivation** | 0.99 | 0.04 | 23.43 | <0.001 | (0.90,1.06) |
| **M11** | **Motivation** | -0.50 | 0.07 | -7.28 | <0.001 | (-0.63,-0.36) |
| **M12** | **Motivation** | 1.05 | 0.04 | 24.73 | <0.001 | (0.96,1.13) |
| **M13** | **Motivation** | 1.05 | 0.04 | 24.77 | <0.001 | (0.97,1.13) |
| **M14** | **Motivation** | 1.15 | 0.05 | 25.5 | <0.001 | (1.06,1.24) |
| **M15** | **Motivation** | 1.14 | 0.05 | 24.8 | <0.001 | (1.05,1.23) |
| **M16** | **Motivation** | 1.14 | 0.04 | 25.95 | <0.001 | (1.04,1.22) |
| **M17** | **Motivation** | 1.12 | 0.04 | 25.66 | <0.001 | (1.03,1.20) |
| **M18** | **Motivation** | 1.12 | 0.05 | 24.57 | <0.001 | (1.02,1.20) |
| **M19** | **Motivation** | 1.05 | 0.05 | 22.51 | <0.001 | (0.96,1.14) |
| **M20** | **Motivation** | -0.44 | 0.06 | -7.6 | <0.001 | (-0.55,-0.32) |
| **M21** | **Motivation** | 0.99 | 0.04 | 22.38 | <0.001 | (0.90,1.07) |
| **M22** | **Motivation** | -0.22 | 0.06 | -3.76 | <0.001 | (-0.33,-0.10) |
| **M23** | **Motivation** | 1.02 | 0.04 | 23.12 | <0.001 | (0.93,1.10) |
| **B1** | **Behavior** | 1.00 |  |  |  |  |
| **B2** | **Behavior** | 1.03 | 0.04 | 26.33 | <0.001 | (0.95,1.10) |
| **B3** | **Behavior** | 1.11 | 0.04 | 26.75 | <0.001 | (1.02,1.18) |
| **B4** | **Behavior** | 0.94 | 0.04 | 22.99 | <0.001 | (0.85,1.01) |
| **B5** | **Behavior** | 0.89 | 0.04 | 21.79 | <0.001 | (0.81,0.97) |
| **B6** | **Behavior** | 0.79 | 0.04 | 18.87 | <0.001 | (0.70,0.86) |
| **B7** | **Behavior** | 1.00 | 0.04 | 25.13 | <0.001 | (0.92,1.07) |
| **B8** | **Behavior** | 0.96 | 0.04 | 23.35 | <0.001 | (0.87,1.04) |
| **B9** | **Behavior** | 1.11 | 0.05 | 23.58 | <0.001 | (1.01,1.19) |
| **B10** | **Behavior** | 1.06 | 0.04 | 24.62 | <0.001 | (0.97,1.14) |
| **B11** | **Behavior** | 1.02 | 0.04 | 23.54 | <0.001 | (0.93,1.10) |
| **B12** | **Behavior** | 1.05 | 0.04 | 23.63 | <0.001 | (0.96,1.13) |
| **B13** | **Behavior** | 1.07 | 0.05 | 23.22 | <0.001 | (0.97,1.15) |
| **B14** | **Behavior** | 0.70 | 0.04 | 15.64 | <0.001 | (0.61,0.79) |
| **B15** | **Behavior** | 0.56 | 0.04 | 12.71 | <0.001 | (0.47,0.64) |
| **B16** | **Behavior** | 0.53 | 0.04 | 12.71 | <0.001 | (0.45,0.61) |
| **N1** | **NPSE** | 1.00 |  |  |  |  |
| **N2** | **NPSE** | 1.10 | 0.05 | 21.68 | <0.001 | (1.00,1.20) |
| **N3** | **NPSE** | 1.11 | 0.05 | 23.55 | <0.001 | (1.01,1.19) |
| **N4** | **NPSE** | 0.94 | 0.05 | 20.26 | <0.001 | (0.84,1.02) |
| **N5** | **NPSE** | 1.04 | 0.06 | 18.17 | <0.001 | (0.92,1.14) |
| **N6** | **NPSE** | 1.14 | 0.06 | 20.6 | <0.001 | (1.03,1.24) |
| **N7** | **NPSE** | 1.09 | 0.06 | 16.94 | <0.001 | (0.96,1.21) |

**Abbreviations:** NPSE, Nursing Professional Self-Efficacy.

**Supplementary Table 8. Model Fit Indices for Latent Profile Analysis Solutions**

| **Model** | **AIC** | **BIC** | **aBIC** | **Entropy** | **LMR** | **BLRT** |
| --- | --- | --- | --- | --- | --- | --- |
| **1** | 14567.538 | 14601.207 | 14575.814 |  |  |  |
| **2** | 13823.363 | 13878.074 | 13836.812 | 0.819 | <0.001 | <0.001 |
| **3** | 13454.774 | 13530.529 | 13473.396 | 0.878 | 0.001 | <0.001 |
| **4** | 13213.070 | 13309.867 | 13236.864 | 0.909 | 0.004 | <0.001 |
| **5** | 13145.819 | 13263.660 | 13174.787 | 0.918 | 0.231 | <0.001 |

**Supplementary Table 9. Class Size and Behavior Scores Across Latent Profile Analysis**

| **Model** | **N (%)** | | | | | **Behavior** |
| --- | --- | --- | --- | --- | --- | --- |
|  | **classes1** | **classes2** | **classes3** | **classes4** | **classes5** | **F (P)** |
| **1** |  |  |  |  |  |  |
| **2** | 261 (52.52) | 236 (47.49) |  |  |  |  |
| **3** | 219 (44.06) | 178 (35.82) | 100 (20.12) |  |  | 337.11 (<0.001) |
| **4** | 29 (5.84) | 99 (19.92) | 174 (35.01) | 195 (39.24) |  |  |
| **5** | 5 (1.01) | 31 (6.24) | 169 (34.00) | 99 (19.92) | 193 (38.83) |  |
