# Supplementary material for: Participation in antimicrobial stewardship among intensive care unit nurses: a cross-sectional study based on the COM-B model
Source: Front Public Health. 2026 Sep 17;14:1922847. doi: 10.3389/fpubh.2026.1922847 (PMC13627333; doi:10.3389/fpubh.2026.1922847)
Supplement: Supplementary file 2 [file Data_Sheet_2.DOCX]

**Questionnaire Number:**

**Dear Participant,**

Hello. We are a nursing research team, and we sincerely invite you to participate in this study. This study aims to investigate ICU nurses’ participation in antimicrobial stewardship (ASP) and its associated factors, identify barriers and facilitators related to capability, opportunity, and motivation, and provide evidence for the development of scientific training programs and intervention strategies, ultimately contributing to improved patient safety and rational antimicrobial use.

Your participation in this study is entirely voluntary. If you agree to participate, you will only need to complete an online questionnaire, which will take approximately 15–20 minutes. The questionnaire includes your demographic information, knowledge and attitudes toward ASP, and actual participation in ASP-related activities. There are no right or wrong answers. Please answer each question truthfully according to your actual situation.

This study is conducted anonymously. Although some basic information will be collected, such as your sex, age, and years of work experience, we promise to keep all information strictly confidential. All data will be used for scientific research purposes only and will not disclose your personal information. This study is a simple questionnaire survey and will not cause any physical or psychological harm. You have the right to be informed about the progress of the study at any time, and you may withdraw from the study at any point without any adverse effect on your work. If you decide to withdraw, you only need to inform us, and your data will be deleted.

Finally, we sincerely thank you for taking the time to support our research despite your busy schedule.

□I have read the above information and agree that the collected data may be used for scientific research.

| **Part 1. General Information** | | |
| --- | --- | --- |
| **Personal Information** |  |  |
| **1.** **Sex:** | a. Male  b. Female |  |
| **2.** **Age: ______ years** | | |
| **Educational and Qualification Background** |  | |
| **3.** **Highest educational attainment:** | a. Secondary vocational diploma  b. Junior college diploma  c. Bachelor’s degree  d. Master’s degree or above | |
| **4.** **Do you hold an ICU specialty nurse certificate?** | a. Yes  b. No | |
| **5.** **Have you received any ASP-related training?** | a. Yes (go to 5.1)  b. No | |
| **5.1** **If yes, what is your cumulative training duration?** | a. <8 hours  b. 8–16 hours  c. 17–40 hours  d. >40 hours | |
| **Professional Background** |  | |
| **6. Years of nursing practice: ______ years** |  | |
| **7. Years of ICU practice: ______ years** |  | |
| **8. Professional title:** | a. Nurse  b. Senior nurse  c. Nurse-in-charge  d. Associate chief nurse or above | |
| **9. Job position:** | a. Clinical nurse  b. Team leader  c. Head nurse  d. Nursing educator | |
| **10. Nurse level:** | a. N0  b. N1  c. N2  d. N3  e. N4 | |
| **11. Employment type:** | a. Permanent position  b. Contract-based  c. Temporary employment | |
| **Work Environment** |  | |
| **12. ICU type:** | a. General ICU  b. Cardiac ICU  c. Respiratory ICU  d. Neurological ICU  e. Emergency ICU  f. Other: ______ | |
| **13. Hospital grade:** | a. Tertiary Grade A  b. Tertiary Grade B  c. Secondary Grade A  d. Other: ______ | |
| **14. Bed-to-nurse ratio in your unit:** | a. 1:2.5–3  b. 1:3–3.5  c. 1:>3.5 | |
| **15. Are there ASP-related policies in your unit?** | a. Yes  b. No  c. Unclear | |

| **Part 2. ICU Nurses’ Participation in ASP Behavior and Influencing Factors Scale** | | | | | |
| --- | --- | --- | --- | --- | --- |
| **Capability** |  |  |  |  |  |
| **Knowledge** |  |  |  |  |  |
| C1. I understand the relevant content of antimicrobial stewardship (ASP) in my hospital, including related policies, regulations, and the roles and responsibilities of nurses. | a. Very knowledgeable | b. Moderately knowledgeable | c. Generally knowledgeable | d. Slightly unknowledgeable | e. Very unknowledgeable |
| C2. I am familiar with the administration timing, routes, and precautions for commonly used antimicrobial agents in the ICU. | a. Very knowledgeable | b. Moderately knowledgeable | c. Generally knowledgeable | d. Slightly unknowledgeable | e. Very unknowledgeable |
| C3. I know how to make a preliminary judgment about the type and severity of infection based on patients’ symptoms, signs, and test results. | a. Very knowledgeable | b. Moderately knowledgeable | c. Generally knowledgeable | d. Slightly unknowledgeable | e. Very unknowledgeable |
| C4. I understand the causes of antimicrobial resistance and its impact on patients. | a. Very knowledgeable | b. Moderately knowledgeable | c. Generally knowledgeable | d. Slightly unknowledgeable | e. Very unknowledgeable |
| **Skills** |  |  |  |  |  |
| C5. I am able to collect pathogen specimens in a standardized manner or guide patients to collect specimens correctly. | a. Never | b. Rarely | c. Sometimes | d. Often | e. Always |
| C6. I am able to identify adverse reactions after antimicrobial use and manage them promptly. | a. Never | b. Rarely | c. Sometimes | d. Often | e. Always |
| C7. I am able to administer antimicrobials correctly according to their pharmacological characteristics (time-dependent or concentration-dependent). | a. Never | b. Rarely | c. Sometimes | d. Often | e. Always |
| C8. I am able to accurately assess whether a patient meets the criteria for switching from intravenous to oral antimicrobial therapy. | a. Never | b. Rarely | c. Sometimes | d. Often | e. Always |
| C9. I have mastered the professional skills required to participate in ASP. | a. Never | b. Rarely | c. Sometimes | d. Often | e. Always |
| **Memory, attention, and decision processes** |  |  |  |  |  |
| C10. In the busy ICU work environment, I am able to continuously pay attention to patients’ antimicrobial use. | a. Never | b. Rarely | c. Sometimes | d. Often | e. Always |
| C11. I am able to apply ASP-related knowledge in actual practice. | a. Never | b. Rarely | c. Sometimes | d. Often | e. Always |
| Behavioral regulation | a. Never | b. Rarely | c. Sometimes | d. Often | e. Always |
| C12. When encountering barriers, I have coping strategies that enable me to continue participating in ASP. | a. Never | b. Rarely | c. Sometimes | d. Often | e. Always |
| C13. I regularly reflect on my performance in ASP and seek ways to improve it. | a. Never | b. Rarely | c. Sometimes | d. Often | e. Always |
| **Opportunity** |  |  |  |  |  |
| **Environmental context and resources** |  |  |  |  |  |
| O1. The ICU where I work provides sufficient time for nurses to participate in ASP activities. | a. Never | b. Rarely | c. Sometimes | d. Often | e. Always |
| O2. I can conveniently access ASP-related resources, such as guidelines, protocols, and decision-support tools. | a. Never | b. Rarely | c. Sometimes | d. Often | e. Always |
| O3. The organizational culture of the hospital supports nurses’ participation in ASP. | a. Never | b. Rarely | c. Sometimes | d. Often | e. Always |
| O4. The unit has sufficient staffing to enable nurses to participate in ASP activities. | a. Never | b. Rarely | c. Sometimes | d. Often | e. Always |
| O5. The hospital information system effectively supports nurses’ participation in ASP. | a. Never | b. Rarely | c. Sometimes | d. Often | e. Always |
| O6. There are clear mechanisms and procedures in the workflow for nurses to participate in ASP. | a. Never | b. Rarely | c. Sometimes | d. Often | e. Always |
| **Social influences** |  |  |  |  |  |
| O7. My colleagues actively participate in ASP activities. | a. Never | b. Rarely | c. Sometimes | d. Often | e. Always |
| O8. ICU physicians support nurses’ participation in ASP-related decision-making. | a. Never | b. Rarely | c. Sometimes | d. Often | e. Always |
| O9. The head nurse encourages us to participate in ASP. | a. Never | b. Rarely | c. Sometimes | d. Often | e. Always |
| O10. The hospital has a multidisciplinary collaboration mechanism (physicians, nurses, pharmacists, and infection control staff) to support ASP. | a. Never | b. Rarely | c. Sometimes | d. Often | e. Always |
| O11. Pharmacists and infection control staff can provide nurses with timely ASP-related support. | a. Never | b. Rarely | c. Sometimes | d. Often | e. Always |
| **Motivation** |  |  |  |  |  |
| **Professional role and identity** |  |  |  |  |  |
| M1. Participation in ASP is an important part of the ICU nurse’s professional role. | a. Never | b. Rarely | c. Sometimes | d. Often | e. Always |
| M2. Participation in ASP can enhance my professional value and sense of professional identity. | a. Never | b. Rarely | c. Sometimes | d. Often | e. Always |
| **Beliefs about capabilities** |  |  |  |  |  |
| M3. Even when work is busy or patients are critically ill, I am still confident in participating in ASP. | a. Never | b. Rarely | c. Sometimes | d. Often | e. Always |
| M4. When I believe that an antimicrobial prescription may be inappropriate, I am confident in raising questions with the physician. | a. Never | b. Rarely | c. Sometimes | d. Often | e. Always |
| M5. If I want to participate in ASP, I am capable of doing so. | a. Never | b. Rarely | c. Sometimes | d. Often | e. Always |
| **Optimism** |  |  |  |  |  |
| M6. I believe that nurses’ participation in ASP can positively affect patient outcomes. | a. Never | b. Rarely | c. Sometimes | d. Often | e. Always |
| M7. I am optimistic about the future implementation of ASP in the ICU. | a. Never | b. Rarely | c. Sometimes | d. Often | e. Always |
| M8. I believe that, through continued effort, nurses can play a greater role in ASP. | a. Never | b. Rarely | c. Sometimes | d. Often | e. Always |
| **Beliefs about consequences** |  |  |  |  |  |
| M9. My participation in ASP can improve patient safety and reduce antimicrobial resistance. | a. Never | b. Rarely | c. Sometimes | d. Often | e. Always |
| M10. My ASP participation behaviors can have a positive effect on infection control. | a. Never | b. Rarely | c. Sometimes | d. Often | e. Always |
| M11. If I question an antimicrobial prescription, it may affect my relationship with the physician. | a. Never | b. Rarely | c. Sometimes | d. Often | e. Always |
| **Reinforcement** |  |  |  |  |  |
| M12. When I participate in ASP, I receive recognition from colleagues or supervisors. | a. Never | b. Rarely | c. Sometimes | d. Often | e. Always |
| M13. Participation in ASP brings me positive feedback and a sense of accomplishment. | a. Never | b. Rarely | c. Sometimes | d. Often | e. Always |
| **Intention** |  |  |  |  |  |
| M14. I intend to participate in ASP activities during my next shift. | a. Never | b. Rarely | c. Sometimes | d. Often | e. Always |
| M15. I plan to actively participate in ASP-related work this week. | a. Never | b. Rarely | c. Sometimes | d. Often | e. Always |
| M16. I have a strong willingness to participate in ASP. | a. Never | b. Rarely | c. Sometimes | d. Often | e. Always |
| M17. I intend to continue increasing my level of participation in ASP in the future. | a. Never | b. Rarely | c. Sometimes | d. Often | e. Always |
| **Goals** |  |  |  |  |  |
| M18. ASP is a priority in my daily nursing practice. | a. Never | b. Rarely | c. Sometimes | d. Often | e. Always |
| M19. I have set specific work goals for my participation in ASP. | a. Never | b. Rarely | c. Sometimes | d. Often | e. Always |
| **Emotion** |  |  |  |  |  |
| M20. I feel anxious when questioning an antimicrobial prescription* (reverse scored). | a. Never | b. Rarely | c. Sometimes | d. Often | e. Always |
| M21. I feel confident when participating in discussions about antimicrobial stewardship. | a. Never | b. Rarely | c. Sometimes | d. Often | e. Always |
| M22. Thinking about participating in antimicrobial stewardship makes me feel stressed. | a. Never | b. Rarely | c. Sometimes | d. Often | e. Always |
| M23. Participating in ASP gives me a sense of accomplishment. | a. Never | b. Rarely | c. Sometimes | d. Often | e. Always |
| **Behavior** |  |  |  |  |  |
| **Assessment and monitoring** |  |  |  |  |  |
| B1. I monitor patients’ therapeutic responses, clinical effectiveness, and adverse reactions after antimicrobial use. | a. Never | b. Rarely | c. Sometimes | d. Often | e. Always |
| B2. I assess whether a patient meets the criteria for switching from intravenous to oral antimicrobial therapy. | a. Never | b. Rarely | c. Sometimes | d. Often | e. Always |
| B3. I pay attention to the duration of antimicrobial therapy and provide timely reminders for review. | a. Never | b. Rarely | c. Sometimes | d. Often | e. Always |
| B4. I supervise the correct timing and route of antimicrobial administration. | a. Never | b. Rarely | c. Sometimes | d. Often | e. Always |
| **Specimen collection** |  |  |  |  |  |
| B5. I collect microbiological specimens in a standardized manner before antimicrobial use. | a. Never | b. Rarely | c. Sometimes | d. Often | e. Always |
| B6. I ensure that the timing and quality of specimen collection meet requirements. | a. Never | b. Rarely | c. Sometimes | d. Often | e. Always |
| B7. I promptly follow up on microbiological culture results. | a. Never | b. Rarely | c. Sometimes | d. Often | e. Always |
| **Communication and collaboration** |  |  |  |  |  |
| B8. When I have concerns about antimicrobial use, I communicate with the physician. | a. Never | b. Rarely | c. Sometimes | d. Often | e. Always |
| B9. I participate in multidisciplinary discussions on antimicrobial use. | a. Never | b. Rarely | c. Sometimes | d. Often | e. Always |
| B10. I consult pharmacists or infection control staff about antimicrobial-related issues. | a. Never | b. Rarely | c. Sometimes | d. Often | e. Always |
| **Patient education** |  |  |  |  |  |
| B11. I educate patients/family members about the correct use of antimicrobials. | a. Never | b. Rarely | c. Sometimes | d. Often | e. Always |
| B12. I explain the risks of antimicrobial resistance to patients/family members. | a. Never | b. Rarely | c. Sometimes | d. Often | e. Always |
| B13. I educate patients/family members on completing the full course of antimicrobial therapy. | a. Never | b. Rarely | c. Sometimes | d. Often | e. Always |
| **Infection control** |  |  |  |  |  |
| B14. I strictly implement standard precautions to prevent healthcare-associated infections. | a. Never | b. Rarely | c. Sometimes | d. Often | e. Always |
| B15. I strictly adhere to hand hygiene protocols to reduce cross-infection. | a. Never | b. Rarely | c. Sometimes | d. Often | e. Always |
| B16. I implement isolation measures for patients with multidrug-resistant organisms. | a. Never | b. Rarely | c. Sometimes | d. Often | e. Always |
| **Open-ended question: In your opinion, what are the main barriers to nurses’ participation in ASP?** | | | | | |

| **Part 3. Nursing Professional Self-Efficacy Scale** | | | | | |
| --- | --- | --- | --- | --- | --- |
| **Care delivery** |  |  |  |  |  |
| N1. I am able to provide safe and effective care in accordance with evidence-based nursing practice. | a. Completely disagree | b. Disagree | c. Uncertain | d. Agree | e. Completely agree |
| N2. I am able to promote health and safety at the community and societal levels. | a. Completely disagree | b. Disagree | c. Uncertain | d. Agree | e. Completely agree |
| N3. I am able to ensure that healthcare delivery consistently meets the highest professional standards. | a. Completely disagree | b. Disagree | c. Uncertain | d. Agree | e. Completely agree |
| N4. I am able to protect patients’ privacy and confidentiality. | a. Completely disagree | b. Disagree | c. Uncertain | d. Agree | e. Completely agree |
| **Professionalism** |  |  |  |  |  |
| N5. I am able to participate in nursing research. | a. Completely disagree | b. Disagree | c. Uncertain | d. Agree | e. Completely agree |
| N6. I am able to collaborate with nursing associations and professional representatives to ensure the best standards of care in practice. | a. Completely disagree | b. Disagree | c. Uncertain | d. Agree | e. Completely agree |
| N7. I am able to report colleagues’ abuse or unethical behavior to the relevant regulatory authorities. | a. Completely disagree | b. Disagree | c. Uncertain | d. Agree | e. Completely agree |

**Thank you again for taking the time to complete our questionnaire. The information you have provided through your responses will be highly valuable to our future work.**

If you have any comments or suggestions regarding this survey study, we would be greatly honored to hear your views.

Comments and suggestions: ______ (optional)

To help ensure that this questionnaire survey can effectively inform future work and facilitate possible follow-up, we would sincerely appreciate it if you would be willing to leave your contact information.

Telephone number: ______ (optional)
